# A Call to Use the Multicomponent Exercise Tai Chi to Improve Recovery From COVID-19 and Long COVID

**DOI:** 10.3389/fpubh.2022.827645

**Published:** 2022-02-28

**Authors:** Juan Pablo Castro, Marie Kierkegaard, Manuel Zeitelhofer

**Affiliations:** ^1^Fundación Neumológica Colombiana, Bogotá, Colombia; ^2^Department of Neurobiology, Care Sciences and Society, Karolinska Institutet, Huddinge, Sweden; ^3^Division of Vascular Biology, Department of Medical Biochemistry and Biophysics, Karolinska Institutet, Stockholm, Sweden

**Keywords:** COVID-19, Tai Chi, multicomponent rehabilitation, quality of life, chronic fatigue, mental health, relaxation response

## Abstract

Approximately 10% of all COVID patients develop long COVID symptoms, which may persist from 1 month up to longer than 1 year. Long COVID may affect any organ/system and manifest in a broad range of symptoms such as shortness of breath, post-exercise malaise, cognitive decline, chronic fatigue, gastrointestinal disorders, musculoskeletal pain and deterioration of mental health. In this context, health institutions struggle with resources to keep up with the prolonged rehabilitation for the increasing number of individuals affected by long COVID. Tai Chi is a multicomponent rehabilitation approach comprising correct breathing technique, balance and neuromuscular training as well as stress- and emotional management. In addition, practicing Tai Chi elicits the relaxation response and balances the autonomic nervous system thus regulating respiration, heart rate, blood pressure and vitality in general. Moreover, Tai Chi has been shown to increase lung capacity, improve cognitive status and mental health, and thereby even the quality of life in diseases such as chronic obstructive pulmonary disease (COPD). Hence, we advocate Tai Chi as potent and suitable rehabilitation tool for post-COVID-19-affected individuals.

## What is Already Known About This Subject?

- Tai Chi is a multicomponent exercise that promotes self-efficacy and empowers affected individuals to actively contribute to their own disease management.
- Tai Chi has been shown to improve life quality and clinical parameters of chronic obstructive pulmonary disease (COPD) and other complex diseases.
- Tai Chi builds strength, improves mobility and balance, and elicits activation of the relaxation response.

## What Are the new Findings?

- Tai Chi is suited for rehabilitation after COVID-19 and treatment of long COVID symptoms.
- Tai Chi potentially improves lung function by counteracting the fibrotic scar formation and may decrease chronic fatigue syndrome by balancing the autonomous nervous system and thereby the risk for development of mental disorders.
- Tai Chi may reduce the economic burden associated with COVID-19 rehabilitation.

## What Are the Recommendations for Policy and Practice?

- We recommend to implement Tai Chi for acute COVID-19 rehabilitation directly after discharge from the hospital and for long COVID.
- The Tai Chi rehabilitation program should last for a minimum of 3 months with twice training for 1 h per week.
- Training by oneself in between the weekly classes should be encouraged.

## Introduction

Physical inactivity increases the risk for modern society diseases such as diabetes and cardiovascular diseases, and likely even susceptibility for infectious diseases such as COVID-19. Recent data have shown that the risk for hospitalization and death due to COVID-19 increases more than 2-fold in physically inactive persons compared to persons performing at least 150 min/week moderate to vigorous physical activity (1). Moreover, one-third of patients suffering from long COVID are still experiencing symptoms such as fatigue, post-exertional malaise, cognitive dysfunction, breathlessness and muscle and joint pain even after 1-year, which results in reduced quality of life (2). Of note, long COVID is very common in young to middle aged persons, a population group engaged in work and family life, potentiating both the social and economic burden associated with the current pandemic. Thus, it is of utmost importance to establish an efficient rehabilitation program to counteract debilitating post COVID symptoms.

## Tai Chi as a Multicomponent Exercise

Tai Chi is a moderate intensity, multicomponent mind-body practice that provides tools for management of chronic diseases, as illustrated by promoting of self-efficacy in patients with chronic obstructive pulmonary disease (COPD) (3). Moreover, it has been shown that Tai Chi has beneficial effects on functional outcomes and quality of life in complex diseases such as cardiovascular, multiple sclerosis (MS), chronic pain and fibromyalgia (4). Tai Chi comprises diaphragmatic breathing technique, balance- and neuromuscular training, postural alignment, stress management and mindfulness. In addition, Tai Chi training teaches the connection of breath and movement and thereby fosters the elicitation of the relaxation response (5). Importantly, the embodied skills learned through practicing Tai Chi may represent a foundation for sustainable self-regulation and self-efficacy, and thereby enable patients to actively contribute to disease management. Finally, potentiation of the practice-associated, long-lasting behavioral changes counts as crucial aspect for management of complex diseases including COVID. Thus, an individual practicing of Tai Chi would in turn significantly reduce the burden for the health care system related to increasing demand for post-COVID rehabilitation.

## Tai Chi Potentially Improves Long COVID Symptoms

A severe COVID-19 disease course can lead to fibrotic changes in the lung (2) that could possibly cause a long-term impairment of the lung function. In COVID patients, spots of inflammation are frequently observed in CT images of the lower lung lobes (6). These patches might make it difficult to breathe during sustained exercise and if unresolved can potentially lead to fibrotic changes. Diaphragmatic breathing learned during Tai Chi encourages air into the lower lobes thus counteracting the inflammatory process. Therefore, exercise becomes an essential rehabilitation tool after acute care and this window of opportunity should be proactively and efficiently used to improve pulmonary function and counteract potential fibrotic changes. It has been shown that Qigong breathing used in Tai Chi leads to a 125 to 145% increase in lung capacity (7). Usually, lung capacity decreases with age due to decreasing tissue elasticity related to fibrotic changes. However, up to 70-year-old Qigong practitioners showed the same lung capacity as 20-year-old non-Qigong practitioners (7).

Recently it has been shown that relaxation response training upregulates genes associated with energy metabolism and mitochondrial function while downregulating genes linked to inflammatory response and stress-related pathways (8). Such boosting of mitochondrial fitness has been speculated to enhance anti-inflammatory effects, which may prevent the occasionally occurring destructive cytokine storm in COVID-19 (9). In addition, it has been demonstrated that Tai Chi modulates the immune response in general by downregulating cytokines such as interleukin-6 that has been implicated in scaring of the lung during COVID-19 (2, 10).

The inflammatory response during COVID-19 is tightly connected with the oxidative stress response leading to accumulation of reactive oxygen species (ROS) and reactive nitrogen species (RNS), by inducing mitochondrial dysfunction and secretion of proinflammatory cytokines. Moreover, ROS also activate transforming growth factor b (TGFb), a key factor for developing lung fibrosis.

While occasional high-intensity physical activity has been shown to promote oxidative stress, regular exercise with moderate intensity (40–59% VO_2max_) decreases the ROS load and DNA damage, respectively, and stimulates key antioxidant enzymes (11). The same form of training intensity is recommended for the elicitation of physiological benefits and the promotion of better health. Thus, the moderate-intensity exercise Tai Chi would represent a suitable training option for improving health status in individuals suffering from post COVID symptoms.

One of the most debilitating long COVID signs is fatigue, which is often occurring in attacks of severe physical and mental tiredness that might result in mental health deterioration such as development of anxiety or depression (12). It has been reported that the fatigue may persist longer than a year post-COVID-19. However, there is an indication that it may last even longer, since 40% of survivors from previous coronavirus outbreaks such as the SARS (severe acute respiratory syndrome) epidemics in 2003 suffered from chronic fatigue and mental illnesses up to 4 years after the disease, which has led to a high unemployment rate and social stigmatization (13).

In addition, a recent study has shown that insomnia, anxiety and depression are very prevalent among the general population during the COVID-19 pandemics with twice as high prevalence as compared to non-pandemic periods and even higher among patients with COVID-19 (14). These observations indicate an urgent need to prevent long-term adverse outcomes associated with insomnia and mental health problems.

Initiation of Tai Chi practice soon after recovering from acute COVID-19 could potentially decrease the risk for developing long-term COVID. Tai Chi improves both blood and energy flow, activates the relaxation response and thus balances the autonomic nervous system (15) and can thereby potentially counteract fatigue and improve cognitive function as well as anxiety and depression (3, 4), all common long COVID symptoms. In addition, the practice has shown to have beneficial effects on cardiovascular health (4), hence, potentially decreasing the risk for COVID-19-associated stroke. Finally, Tai Chi has been shown to improve exercise capacity, balance and posture/neuromuscular control, which may occur in COVID-19 patients (3, 4).

## A Call for Action

Tai Chi potentially improves lung function by counteracting the fibrotic scar formation and decreasing long-term COVID fatigue and thereby the risk for development of mental disorders. Moreover, the practice is at the same time likely to increase muscular strength, mobility and vitality, which should in turn empower the individuals affected by COVID-19 to actively contribute to their recovery. Hence, implementation of Tai Chi in rehabilitation of COVID-19-affected individuals, for both short- and long-term disease courses, can be highly recommended.

## Author Contributions

MZ and JC designed the manuscript. MZ wrote the manuscript together with JC and MK. All authors contributed to the article and approved the submitted version.

## Conflict of Interest

JC is holding rehabilitation classes at Fundación Neumológica Colombiana for COPD and post COVID patients. In addition, he has his own Tai Chi classes. The remaining authors declare that the research was conducted in the absence of any commercial or financial relationships that could be construed as a potential conflict of interest.

## Publisher's Note

All claims expressed in this article are solely those of the authors and do not necessarily represent those of their affiliated organizations, or those of the publisher, the editors and the reviewers. Any product that may be evaluated in this article, or claim that may be made by its manufacturer, is not guaranteed or endorsed by the publisher.
